# Concurrent Negative-Pressure Pulmonary Edema (NPPE) and Takotsubo Syndrome (TTS) after Upper Airway Obstruction

**DOI:** 10.1155/2019/5746068

**Published:** 2019-06-09

**Authors:** Evan Harmon, Sebastian Estrada, Ryan J. Koene, Sula Mazimba, Younghoon Kwon

**Affiliations:** ^1^University of Virginia Health System, Charlottesville, VA, USA; ^2^Cleveland Clinic Foundation, Cleveland, OH, USA

## Abstract

Upper airway obstruction is a potentially life-threatening emergency often encountered in the acute care, perioperative, and critical care settings. One important complication of acute obstruction is negative-pressure pulmonary edema (NPPE). We describe two cases of acute upper airway obstruction, both of which resulted in flash pulmonary edema complicated by acute hypoxic respiratory failure. Though NPPE was suspected, these patients were also found to have Takotsubo syndrome (TTS). Neither patient had prior cardiac disease, and both subsequently had a negative ischemic workup. Because TTS is a condition triggered by hyperadrenergic states, the acute airway obstruction alone or in combination with NPPE was the likely explanation for TTS in each case. These cases highlight the importance of also considering cardiogenic causes of pulmonary edema in the setting of upper airway obstruction, which we suspect generates a profound catecholamine surge and places patients at increased risk of TTS development.

## 1. Introduction

NPPE is a rare but well-described complication of upper airway obstruction and has been of particular interest to clinicians and researchers alike as it represents one of the few noncardiogenic causes of flash pulmonary edema. However, acute obstruction may also place patients at higher risk of cardiac complications which can cause or exacerbate pulmonary edema. One such complication is the development of Takotsubo syndrome (TTS), which is underdiagnosed, particularly in the perioperative and critical care settings, given that it is commonly mistaken for an acute coronary syndrome with subsequent ischemic cardiomyopathy. Though both NPPE and TTS individually have been well-described, there is a paucity of reports describing the sudden, concurrent development of *both* NPPE and TTS in patients suffering acute upper airway obstruction.

## 2. Case Descriptions

### 2.1. Case 1

A 39-year-old woman with a history of borderline personality disorder, bipolar disorder, and depression presented to the emergency department (ED) intubated after a suicide attempt by hanging. Her partner at the bedside estimated the patient had been hanging for roughly ten minutes prior to being discovered and also suspected concurrent overdose on home alprazolam. Admission vital signs and exam were remarkable for HR 112 bpm, BP 90/60 mmHg, pupils 6 mm and reactive to light, GCS 3, and neck erythema.

**Figure 1:**
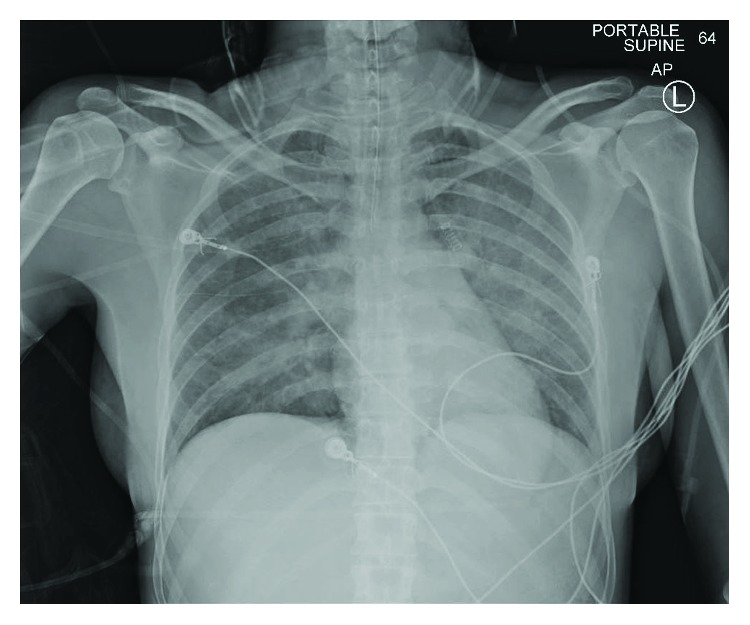
Chest radiograph demonstrating diffuse pulmonary edema upon admission to the emergency department.

The initial laboratory workup was notable for white blood cell (WBC) count 18.6 K/*μ*L, troponin 0.24 ng/mL (peak of 1.78 ng/mL), and arterial blood gas (ABG) on FiO_2_ 100% demonstrating pH 7.201, pCO_2_ 36 mmHg, pO_2_ 231.6 mmHg, bicarbonate 13.8 mmol/L, anion gap 8 mmol/L, and lactate 0.86 mmol/L. Electrocardiogram demonstrated sinus tachycardia with no ST-segment or T-wave changes. Trauma imaging in the ED included contrasted tomography (CT) of the head without contrast, CT cervical spine, and CT angiography of the neck, which were negative for cervical spine injury, cervical artery dissection, or brain hemorrhage or infarct. However, chest X-ray (CXR) demonstrated diffuse, bilateral pulmonary edema (Figure 1). The patient was also found to be hypotensive and required vasopressor support. She was transferred to the intensive care unit (ICU) for further management of NPPE complicated by acute hypoxic respiratory failure, severe nonanion gap metabolic acidosis, and presumed type II non-ST-segment elevation myocardial infarction (NSTEMI).

**Figure 2:**
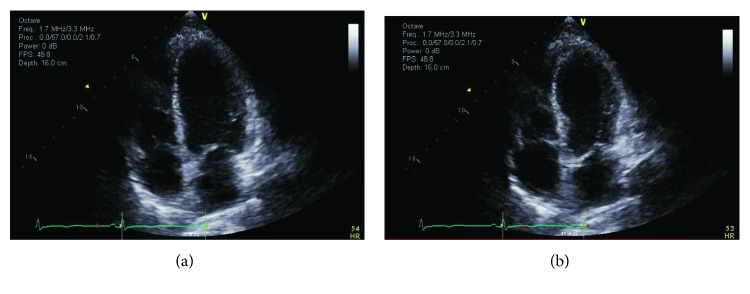
Echocardiographic images demonstrating end-diastole (a) and end-systole (b). Basal and apical thickening with sparing of the midchamber segments indicates a midventricular variant of TTS.

Shortly after arrival to the ICU, transthoracic echocardiogram (TTE) was obtained which demonstrated a left ventricular (LV) ejection fraction (LVEF) 40-45% and LV wall-motion abnormality consistent with midventricular form of Takotsubo syndrome (TTS, Figure 2). Intravenous diuresis with furosemide was administered with satisfactory response. Additionally, the patient's metabolic derangements corrected with supportive measures, sedation and vasopressor support were weaned, and she was successfully extubated within 48 hours of admission without any adverse neurologic sequelae. She was successfully started on metoprolol tartrate 25 mg twice daily with goal conversion to succinate formulation and uptitration to therapeutic effect as hemodynamically tolerated. The patient was transferred for continued inpatient psychiatric evaluation and management. Follow-up echocardiogram three months after initial TTE demonstrated full recovery of LV function.

### 2.2. Case 2

A 64-year-old man without history of cardiac disease was admitted for debridement of anterior mandibular osteomyelitis. Preoperative cardiopulmonary assessment was unremarkable, and the patient tolerated the procedure well. Shortly after extubation, he unexpectedly demonstrated labored breathing with inspiratory stridor. Laryngospasm was visualized by direct laryngoscopy, and the patient ultimately required reintubation. Appropriate endotracheal tube placement was confirmed by chest rise and CO_2_ monitor, yet the patient was found to have oxygen saturation of 90% on 100% FiO_2_. He subsequently became severely hypotensive requiring vasopressor support and ICU admission.

Upon arrival, pulmonary auscultation revealed bilateral rales, and frothy secretions were suctioned via the endotracheal tube. The initial workup was most significant for troponin elevated to 1.76 ng/mL (normal < 0.021 ng/mL), electrocardiogram with new, diffuse, deep T-wave inversions, CXR demonstrating flash pulmonary edema, and bedside TTE demonstrating severely reduced LVEF of 20% with diffuse akinesis involving the mid-to-apical segments of the LV with basal sparing. Right ventricular function was normal. Coronary angiogram was negative for acute coronary syndrome or any significant obstructive coronary artery disease.

These findings suggested TTS as the etiology of patient's acute cardiogenic shock. He responded well to intravenous diuretic therapy and was successfully extubated and weaned from all inotropic/vasopressor support within five days of ICU admission. This clinical improvement corresponded with radiographic resolution of his pulmonary edema. Seven days after initial presentation, repeat TTE demonstrated EF improvement to greater than 40% with distal anteroseptal periapical and distal inferoseptal hypokinesis and akinesis. At ambulatory follow-up appointment two months after hospital discharge, he was found to be asymptomatic with ECG normalization.

## 3. Discussion

Negative-pressure pulmonary edema (NPPE) is an increasingly described life-threatening emergency in the perioperative and critical care settings. Upper airway obstruction is the fundamental etiology of this phenomenon, with upper airway infection, tumor, and laryngospasm thought to be the most common causes [1]. Though a myriad of etiologies have been associated with NPPE since first reported in the pediatric literature over forty years ago [2], a unifying pathophysiological process has been elusive to date. Overall, it is thought that inspiratory effort against an obstructed glottis results in increased hydrostatic forces in the pulmonary microvasculature. This disruption of Starling forces, combined with profoundly negative intrathoracic airway pressures, results in fluid extravasation into the interstitium and subsequent flash pulmonary edema.

However, we describe two cases of NPPE, one due to asphyxiation and the other laryngospasm, which were further complicated by Takotsubo syndrome (TTS), an acute heart failure syndrome typically affecting women over the age of 50. It is characterized by apical or midventricular LV dysfunction with new ECG and/or serum cardiac biomarker abnormalities but without obstructive coronary disease and/or plaque rupture by coronary angiography [3]. To our knowledge, these cases represent two of only four total reports of concurrent NPPE and TTS [4, 5]. A number of pathophysiologic mechanisms of TTS have been proposed which typically revolve around a sudden and profound catecholamine release, including catecholamine-induced multivessel epicardial spasm and cyclic AMP-mediated intracellular calcium overload [6, 7]. Each of these mechanisms directly induce myocyte injury, though the role of the former in TTS propagation may be less relevant given that TTS may be precipitated by direct beta-agonism. Specifically, increasing evidence suggests elevated levels of peripheral epinephrine alter *β*2-adrenergic receptor signaling by inducing a switch from stimulatory to inhibitory G protein subcellular signaling [8].

**Figure 3:**
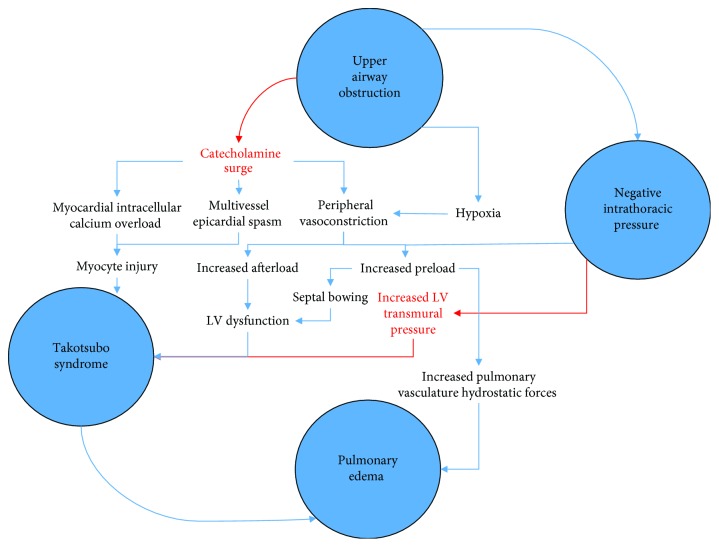
Proposed pathophysiologic diagram of NPPE and TTS. Notice multiple shared processes between the two which are thought to contribute to pulmonary edema following upper airway obstruction (note: red indicates proposed mechanisms).

It is precisely this catecholaminergic surge which we suspect serves as the pathophysiologic link between NPPE and TTS (Figure 3). Attempted inhalation against a fixed obstruction in the form of a closed glottis results in both negative intrathoracic pressure *and* what is likely a dramatic catecholaminergic surge. This hyperadrenergic state not only contributes to both direct and indirect myocyte injuries as previously described but also results in peripheral vasoconstriction which serves to increase both preload and afterload. A sudden increase in right ventricular preload has two primary effects: first, pulmonary vasculature volume increases, contributing to increased hydrostatic forces. Second, greater venous return to the RV may result in septal bowing with resultant LV outflow tract obstruction, thereby diminishing LV EF. Additionally, elevated peripheral sympathetic activity results in arterial vasoconstriction, and this increased afterload places undue strain on the LV. Finally, negative intrathoracic airway pressure itself has been demonstrated to increase LV transmural pressure, further diminishing LV function.

Thus, upper airway obstruction may compromise LV function by at least four mechanisms: increased RV preload with resultant septal bowing, increased afterload via catecholamine-induced vasoconstriction, increased LV transmural pressure via the sudden generation of negative intrathoracic pressure, and rarely, as in the cases reported here, catecholamine-induced cardiomyocyte damage resulting in TTS. It should also be noted that increasing evidence has implicated the shedding of endovascular glycocalyx, an important regulator of vascular permeability, as a critical pathophysiologic mechanism at the cellular level by which TTS may induce pulmonary edema [10]. The summative effect of these physiologic insults is almost certain to be cardiogenic shock, which worsens pulmonary vasculature congestion and exacerbates NPPE. The final pathway of these processes is flash pulmonary edema with acute respiratory distress and often failure, which is accompanied by further catecholamine production, promoting a complex, life-threatening pathophysiologic cycle.

Therefore, flash pulmonary edema complicated by acute hypoxic respiratory failure encountered in the setting of upper airway obstruction may be due to both noncardiogenic *and* cardiogenic factors. This is especially important to remember in the perioperative period, when cardiac dysfunction due to TTS may be misattributed to infarction. We recommend emergent cardiac evaluation including troponin, ECG, and bedside TTE. In patients with troponinemia, newly reduced or worsened EF, wall-motion abnormalities, and/or ischemic changes on ECG, a more invasive workup including coronary angiogram should be considered. A profound elevation in BNP or NT-proBNP disproportionate to troponin elevation may also serve as an indicator of TTS rather than evolving myocardial infarction. If no ischemic etiology is identified, diagnosis may ultimately be confirmed by the visualization of diffuse, nonlocalizing ventricular edema on cardiac MRI. Management should focus on intravenous diuresis, positive-pressure ventilation, and hemodynamic support. Future research should determine what prognostic significance, if any, should be ascribed to the concurrent development of NPPE with TTS in patients with acute upper airway obstruction. Anecdotal evidence derived from the cases presented here and those reported elsewhere suggests that full recovery of LVEF can be expected with proper management, though the aforementioned pathophysiologic changes in LV structure may persist.

## 4. Conclusion

NPPE is a relatively rare but well-described life-threatening complication of upper airway obstruction. However, TTS is one of multiple cardiogenic etiologies which may also be contributing to flash pulmonary edema in this patient population. Though the precise mechanisms by which each of these pathologies occur have not been delineated, we suspect upper airway obstruction alone or in combination with NPPE generates a profound catecholamine surge which may be a key in triggering TTS. In select patients, ischemic evaluation may be warranted. Despite rapid and dramatic cardiopulmonary compromise, these patients appear to quickly achieve full recovery with proper management, though further research is necessary to definitively determine long-term prognosis.
